# *Spirocerca lupi* Proteomics and Its Role in Cancer Development: An Overview of Spirocercosis-Induced Sarcomas and Revision of Helminth-Induced Carcinomas

**DOI:** 10.3390/pathogens10020124

**Published:** 2021-01-26

**Authors:** Catalina Porras-Silesky, María José Mejías-Alpízar, Javier Mora, Gad Baneth, Alicia Rojas

**Affiliations:** 1 Laboratory of Helminthology, Centro de Investigación en Enfermedades Tropicales, University of Costa Rica, 11501-2060 San José, Costa Rica; catalinaps.cp@gmail.com (C.P.-S.); mariajose911@gmail.com (M.J.M.-A.); javierfrancisco.mora@ucr.ac.cr (J.M.); 2 Koret School of Veterinary Medicine, The Hebrew University of Jerusalem, Rehovot 7610001, Israel; gad.baneth@mail.huji.ac.il

**Keywords:** *Spirocerca lupi*, spirocercosis, proteomics, cancer, helminth, excretory and secretory products

## Abstract

*Spirocerca lupi* is a parasitic nematode of canids that induces a myriad of clinical manifestations in its host and, in 25% of infections, leads to the formation of sarcomas. The description of the protein composition of the excretory and secretory products (*Sl*-ESP) of *S. lupi* has shed light on its possible interactions with the host environment, including migration within the host and mechanisms of immunomodulation. Despite this, the process by which *S. lupi* induces cancer in the dog remains poorly understood, and some hypotheses have arisen regarding these possible mechanisms. In this review, we discuss the role of specific ESP from the carcinogenic helminths *Clonorchis sinensis, Opisthorchis viverrini* and *Schistosoma haematobium* in inducing chronic inflammation and cancer in their host’s tissues. The parasitic worms *Taenia solium, Echinococcus granulosus, Heterakis gallinarum, Trichuris muris* and *Strongyloides stercoralis*, which have less-characterized mechanisms of cancer induction, are also analyzed. Based on the pathological findings in spirocercosis and the mechanisms by which other parasitic helminths induce cancer, we propose that the sustained inflammatory response in the dog´s tissues produced in response to the release of *Sl*-ESP homologous to those of other carcinogenic worms may lead to the malignant process in infected dogs.

## 1. *Spirocerca lupi* Overview 

*Spirocerca lupi* is a parasitic nematode of dogs (Spirurida: Spirocercidae) and the causative agent of the severe disease spirocercosis [1], which produces a myriad of clinical signs but, most importantly, induces the formation of fibrosarcoma and osteosarcoma in the esophagi of dogs [2]. *S. lupi* has been detected mainly in tropical and subtropical regions of the world, with some geographical areas showing a marked seasonality and most cases occurring in winter [3]. In addition, reports from temperate locations such as Hungary and Italy have increased in the last decade [4,5], confirming the threat of infection in areas where the disease has not been detected previously. This suggests that spirocercosis has become endemic in certain geographical areas due to climate change, urbanization, pet travel and the possible dissemination of dung beetle vectors [6].

Domestic dogs act as definitive hosts in the life cycle of *S. lupi*, whereas other wild canid species can also become infected with this parasite [2]. Definitive hosts become infected with the ingestion of intermediate or paratenic hosts containing encapsulated and infective third-stage larvae (L3). L3 larva excysts in the stomach and penetrates the gastric mucosa, reaching the gastric arteries and thoracic aorta, where it molts to a fourth-stage larva (L4) and migrates across the intima of the aorta to the esophagus [2,7]. *S. lupi* worms induce the formation of esophageal nodules characterized by a nipple-like orifice in the mucosa, through which females release embryonated and infective eggs [3], containing first-stage larvae (L1). Then, coprophagous beetles of the Scarabaeidae family become infected with the ingestion of eggs from feces and foster larval development under specific physical and chemical conditions [8]. Paratenic hosts, such as rabbits, lizards, hedgehogs, rodents, poultry and wild birds, can become infected by the ingestion of dung beetles with L3 [2,7]. L3 re-encysts in the paratenic host tissues, mainly in the gastric wall [9], causing edema, necrosis and inflammation with activated macrophages, lymphocytes, eosinophils and collagen fibers [10]. Some reports in experimentally infected rabbits indicate that L3 can also migrate to the aorta, causing fibrotic and necrotic lesions, accompanied by strong inflammatory reactions [10].

The clinical presentation of spirocercosis in dogs depends on the stage of the disease, which can progress from an early-inflammatory esophageal nodule or pre-neoplastic nodule to a fibro-, osteo- or chondrosarcoma [2]. The prepatent period of esophageal spirocercosis from initial infection to maturation into adults is approximately 3 to 6 months, in which eggs can be observed in feces or, less frequently, in the dog´s vomitus [7]. The most common clinical signs during the early stage of the disease are the regurgitation of food and vomiting, weight loss, weakness, pyrexia, melena and hypersalivation [3,6,7,11]. In addition, *S. lupi* produces hypertrophic osteopathy and aortic lesions, leading to thromboembolism, aortic rupture and sudden death [3]. Moreover, aberrant migrations to thoracic, gastrointestinal [12,13] and spinal [14] tissues can also occur. Pathognomonic lesions of spirocercosis include aortic scarring with aneurysms, thoracic spondylitis and caudal esophageal nodules [7,15,16]. 

**Figure 1:**
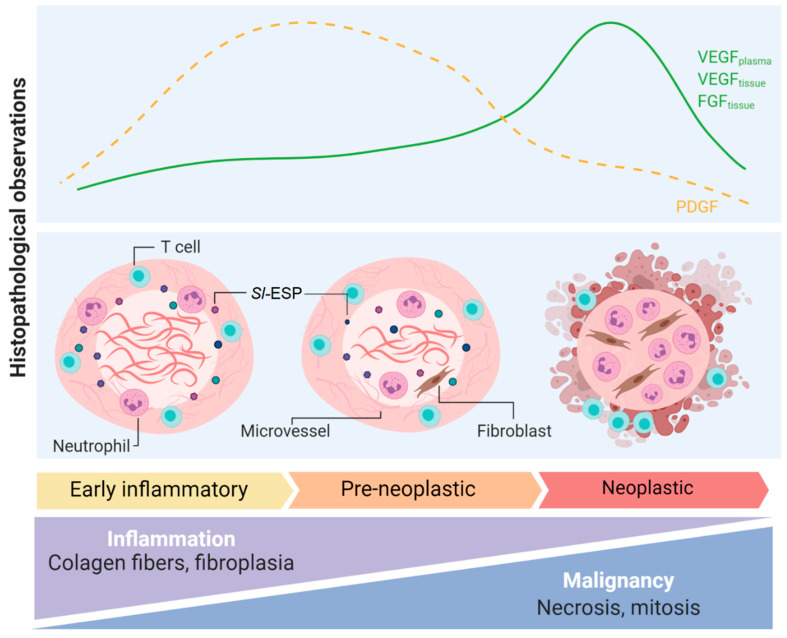
Pathogenic mechanisms observed in *S. lupi*-induced nodules in the dog’s esophagus. Histopathological observations, blood and tissue growth factors and overall changes in early-inflammatory, pre-neoplastic and neoplastic esophageal spirocercosis nodules. FGF: fibroblast growth factor; PDGF: platelet-derived growth factor; *Sl*-ESP: excretion–secretion products derived from *S. lupi*; VEFG: vascular endothelial growth factor. This figure was created using Biorender.com.

*S. lupi* has been associated with the formation of sarcomas in canids, with reports of the malignant transformation of esophageal nodules in approximately 25% of infected dogs, with osteosarcoma and fibrosarcoma comprising the most common diagnoses [6,17,18]. Other types of *S. lupi*-induced sarcomas are chondrosarcoma [19] and undifferentiated sarcoma [7]. The metastasis of these sarcomas to various organs such as the lungs, kidneys, stomach, spleen, heart and tongue has been frequently reported [20]. The analysis of non-neoplastic nodules reveals that adults, eggs and migratory tracts are observed at a higher percentage in these lesions compared to neoplastic nodules [21]. Due to the differences in cellular infiltrates and overall tissue findings, non-neoplastic tumors are divided in two phases: early inflammatory, composed of lymphoplasmic inflammation, inactive fibrocytes and a great deposition of collagen, and pre-neoplastic, with activated and atypical fibroblasts, reduced collagen and lymphoplasmic inflammation, with a high mitosis index. Moreover, a third neoplastic phase is characterized by neutrophil infiltrates, fibrin deposition, mitotic cells, multinucleated giant cells and necrosis (Figure 1) [2,21]. 

Different growth factors and cytokines have been studied as blood markers of neoplastic transformation and also to increase the understanding of the pathological processes underlying malignant spirocercosis. Dogs with malignant esophageal nodules have significantly increased levels of plasmatic interleukin-8 (IL-8) [22]. IL-8, involved in tumor progression in Epstein–Barr virus-induced carcinoma [23], may be released by activated fibroblasts present in pre-neoplastic nodules, working as a neutrophil chemoattractant during *S. lupi* infection. These neutrophils will subsequently release more IL-8, promoting sustained inflammation and possibly chronic tissue damage that might lead to neoplasia [22]. Interestingly, significant differences in the proinflammatory cytokine interleukin-18 (IL-18) were reported in the same study. Dogs with non-neoplastic nodules showed the highest IL-18 concentrations in plasma, followed by the control group and the neoplastic group with the lowest IL-18 concentrations [22]. IL-18 is a proinflammatory cytokine associated with the production of interferon gamma (IFN-γ) from lymphocyte T cells and natural killer (NK) cells promoting T helper type 1 (Th1) responses. However, in the context of inflammation and tumor development, IL-18’s function depends on the inflammatory milieu and its interaction with other immune mediators [24]. 

The expression of vascular endothelial growth factor (VEGF), platelet-derived growth factor (PDGF) and fibroblast growth factor (FGF) (Figure 1) have been analyzed in canine spirocercosis. VEGF and FGF significantly increased as the nodule progressed from early-inflammatory to neoplastic [25], whereas PDGF was detected in higher amounts in pre-neoplastic and early non-neoplastic nodules [25]. Furthermore, circulating VEGF in plasma and serum was significantly higher in dogs with neoplastic nodules compared to dogs with non-neoplastic nodules [26]. This suggests that VEGF might play a role in inducing angiogenesis in malignant nodules. However, microvessels were of lower abundance in neoplastic nodules compared to early-inflammatory and pre-neoplastic nodules, as shown for other soft tissue sarcomas [27]. This might be associated with changes in other immune mediators during progression to neoplastic nodules. Besides VEGF production, angiogenesis requires other growth factors and inflammatory cytokines such as interleukin 1β (IL-1β), tumor necrosis factorα (TNF-α) and PDGF, which as mentioned above, were reduced in neoplastic nodules [28].

Cell infiltrates in *S. lupi*-induced esophageal nodules are mostly of the myeloid lineage [21]. Neutrophils gather in pockets around nematodes from non-neoplastic nodules or in necrotic–ulcerative areas in neoplastic lesions [29]. Lymphocytes usually show a focal/multifocal distribution in the periphery of the nodules, with CD4+ T cells more abundant than B cells. This different infiltration pattern might be associated with the low vascularization level of the nodules; therefore, lymphoid cells remain at the periphery whereas myeloid cells with higher tissue motility predominantly infiltrate the nodule. Interestingly, low amounts of FoxP3+ regulatory T (Treg) cells are found in all nodule stages, whereas these cells are increased in neighboring lymph nodes. This suggests that “homing” of FoxP3+ cells from lymph nodes to esophageal nodules is affected by an unknown mechanism. FoxP3+ cells are highly abundant in different tumors due to their role in suppressing antitumor immunity and hindering protective immunosurveillance [30]. The apparent absence of FoxP3+ cells in *S. lupi* neoplastic nodules can be explained by the presence of other types of Treg cells or subtypes of myeloid suppressor cells that could be involved in the malignant process during spirocercosis [29]. 

## 2. *Spirocerca lupi* Proteomics

Excretory and secretory products (ESP) are molecules released by helminths into the host environment by two different mechanisms: the active secretion of functional products exported through secretory pathways or the excretion of parasitic waste products [31,32]. Some examples of ESP include proteases, protease inhibitors, lectins and other molecules, whose abundance depends on the stage of the parasite’s life cycle [32,33]. These molecules play different roles in host–pathogen interactions, including immunoregulation [31], cell migration, adhesion, proliferation, differentiation and invasion [33].

Two independent studies have researched the composition of *S. lupi*-derived ESP (*Sl*-ESP) [34,35]. In one study, nine proteins were detected by liquid chromatography mass spectrometry (LC-MS), and only three had appended annotations [34]. Moreover, *in vitro* analyses of the ESP with mouse fibroblasts did not show mitogenic effects, suggesting that *Sl*-ESP alone do not have a direct influence on these cells [34]. In the second study of the *S. lupi* secretome, 838 different peptides were obtained from L3, L4 and adult stages of the parasite, corresponding to 211 proteins, 171 with appended annotations and 40 without characterization [35]. Forty-four proteins were shared between stages, involved mainly in carbohydrate, protein and nucleic acid metabolism, such as glyceraldehyde 3-phosphate dehydrogenase, triosephosphate isomerase and serine–threonine phosphatases [35]. Furthermore, proteins involved in immunoregulation in other helminth infections such as the retinoid fatty acid binding protein and galectin, or in detoxifying reactive-oxygen species (ROS) such as glutathione S-transferase, thioredoxin or peroxiredoxin, were also common in all the *S. lupi* stages. Interestingly, 49 molecules related to interaction and remodeling with the extracellular matrix were detected only in L4, such as cuticlin-1, basement membrane proteoglycan, mua-3 cell transmembrane adhesion receptor and qua-1. These proteins might have a role in migration through dog tissues and simultaneous molting to adult stages [35]. L3 uniquely secreted cytoskeletal proteins such as alpha actinin and dynein light chain 1, and were enriched in lipid metabolism molecules such as acyl coenzyme A dehydrogenase protein and enoyl coenzyme A hydratase [35]. These results indicate that *Sl*-ESP are highly dynamic and are modified at different levels according to the evolutionary stage of the parasite [33,35].

The role of *Sl*-ESP in the development of malignant nodules remains poorly understood, since growth factors or pro-oncogenic molecules have not been detected in the L3, L4 or adult stages [35]. Strikingly, annexin 6, a molecule involved in other normal processes of the dog, is one of the potential candidates that was found and suggested to play a role in the pathogenesis of *S. lupi*-induced fibrosarcoma and osteosarcoma [35]. Annexin 6 was expressed mainly in L4 and adults, the stages that interact with the dog host, and its overexpression in humans has been correlated with cervical cancer [36] and lymphoblastic leukemia [37]. The specific function of this protein in *S. lupi* oncogenesis is unknown and, thus, requires additional exploration. Additionally, other *Sl*-ESP commonly found in other nematodes such as calreticulin, heat shock protein 70 (HSP70) and heat shock protein 90 (HSP90) are potential carcinogenic inducers acting extracellularly on epithelial, stromal and immune cells [38,39]. Other *Sl*-ESP with intracellular oncogenic potential include 14-3-3 protein, elongation-factor-1 alpha, HSP70 and HSP90 [39,40,41]. 

Moreover, the low similarity of some *Sl*-ESP to ESP found in other nematode databases suggests that these proteins could have other functions in processes not described yet, including carcinogenesis, requiring further investigation. The analysis of other helminth-induced carcinomas might shed light on the possible molecules or pathways that might be involved in *S. lupi*-associated esophageal sarcomas. 

## 3. Proteins Involved in Carcinogenesis in Other Helminthiases

Three species of human parasitic trematodes or flatworms have been classified by the International Agency for Research on Cancer (IARC) as group I carcinogens, namely, *Clonorchis sinensis, Opisthorchis viverrini* and *Schistosoma haematobium*. Other infectious agents classified as group I carcinogens include hepatitis B and C viruses that induce hepatic cancer, and *Helicobacter pylori*, linked to gastric cancer [42]. Furthermore, the fluke *Schistosoma japonicum* has been classified as a group 2B carcinogen, whereas *Schistosoma mansoni* and *Opisthorchis felineus* are classified as group 3 carcinogens. Helminth-associated cancer can be reduced or prevented by educational and sanitation practices. Therefore, the study of the processes leading to malignancy has public health connotations. Moreover, the chronic nature of helminth infections not only increases the risk of developing cancer in infected humans, but also perpetuates the dissemination of the infectious stages to healthy populations [43].

### 3.1. Clonorchis sinensis

*C. sinensis* is a liver fluke endemic in Asian countries and transmitted to humans by the ingestion of raw or undercooked fish carrying metacercariae [44]. This parasite can induce the formation of hepatocarcinoma or cholangiocarcinoma (CCA). *C. sinensis* colonization causes mechanical damage to bile epithelia that results in a severe inflammatory response in biliary epithelial cells, hyperplasia, metaplasia and, finally, periductal fibrosis [45]. *C. sinensis*-derived ESP (*Cs*-ESP), including secretory phospholipase A (2) [46], lysophospholipase [47], fructose-1,6-bisphosphatase [48] and the ferritin heavy chain protein (*Cs*FHC) [49] (Table S1), directly activate human hepatic stellate cells and play key roles in the development of liver fibrosis and the production of collagen [49,50,51]. Chronic infection promotes the production of increased amounts of fibrous tissue, which may encase proliferating glands, causing cholangiofibrosis [45].

*Cs*-ESP are highly immunogenic, stimulate inflammatory reactions, promote host cell proliferation and suppress apoptosis in the biliary epithelia [52,53] and, thus, are instrumental in the induction of CCA [45,52,54]. It has been observed that Transforming growth factor-beta (TGF-β) receptor interacting protein 1, legumain and growth factor binding protein 2 stimulate the secretion of the proinflammatory IL-1β, interleukin 6 (IL-6) and TNF-α cytokines in hepatic cells [55]. In addition, *Cs*FHC triggers the production of oxygen and nitrogen free radicals in cells, leading to the expression of proinflammatory cytokines [49]. Interestingly, *Cs*-ESP also include ROS-degrading molecules, such as glutathione S-transferase, thioredoxin peroxidase, myoglobin and a number of cysteine proteases [51,52], which are useful for evading neutrophil-derived ROS. Moreover, *Cs*-ESP induce the expression of nuclear factor kappa B (NF-kB) and TNF-α via Toll-like receptor 4 stimulation [56,57], suggesting an active role of these molecules in the immunopathological response of the host [55]. Annexin B30 was detected in *Cs*-ESP and triggers strong interleukin 10 (IL-10) production in splenocytes, which might affect the immune response host during infection [58].

A homologue of human granulin (*Cs*GRN) has been identified in the tegument and testes of adult *C. sinensis* worms and also deposited in the liver tissues of infected mice [53,59]. *Cs*GRN may promote carcinoma progression by inducing angiogenesis, insensitivity to apoptosis, tumor invasion and anchorage dependence [59,60]. In addition, the overexpression of *Cs*GRN increases the expression of vimentin, N-cadherin and β-catenin, and decreases zonula occludens 1 (ZO-1), indicating that this protein is involved in promoting cell–cell adhesion for metastases [59].

### 3.2. Opisthorchis viverrini

The liver fluke *O. viverrini* represents a public health problem in Southeast Asia [61,62,63], and similar to *C. sinensis,* the transmission of *O. viverrini* to humans occurs after the ingestion of uncooked fish [54]. The cancer associated with opisthorchiasis results from the combination of several factors, including mechanical damage to bile ducts by the worms, chronic inflammation and direct effects of ESP released by the parasite (*Ov*-ESP) [54,61,64]. 

*O. viverrini* survives the hostile environment of bile ducts, aided by *Ov*-ESP originated from the worm’s tegument [65], which promote cell proliferation and IL-6 secretion [66]. High levels of IL-6 have been associated with oxidative stress and DNA damage, which also lead to fibrosis and CCA [63,64]. The malignant transformation during opisthorchiasis is associated with the constant feeding on and destruction of bile tissues by the parasites [62,63]. Special focus has been granted to the secreted antioxidant proteins thioredoxin and peroxiredoxin, and a granulin-like growth factor, *Ov*-GRN-1 (Table S1), as effectors of pro-oncogenic processes [67]. It has been demonstrated that thioredoxin downregulates apoptotic genes; upregulates anti-apoptosis-associated genes, such as caspases 3, 8 and 9 [68]; and has growth-factor properties [69,70], whereas peroxiredoxin inhibits apoptosis induced by hydrogen peroxide [64]. *Ov*-GRN-1 has been shown to accelerate wound healing in mouse models [64,71] and stimulate angiogenesis at nanomolar concentrations [72,73], which can promote metastasis and tumor progression [53,71].

A close relative of *O. viverrini*, *Opisthorchis felineus*, has also been regarded as a potential human carcinogen. Epidemiological data associate infection with this fluke with severe hepatobiliary disease [74] and regard it as a risk factor for CCA [75]. However, the mechanisms by which this parasite leads to these pathologies remain unknown.

### 3.3. Schistosoma haematobium

*S. haematobium* is transmitted by the skin penetration of free cercariae present in lakes and rivers and is the only human schistosome directly associated with cancer [76]. This parasitic trematode is located inside bladder venules, contributing to irritation and fibrosis that leads to squamous cell bladder cancer [45]. The incidence of squamous cell bladder cancer is significantly higher in areas with *S. haematobium* infection, such as the Middle East and Africa [77]. Inflammatory and immune responses during schistosomiasis triggered by egg deposition elicit a granulomatous response around the eggs [76] and lead to hematuria and pre-carcinogenic changes in endodermal organs [78], such as bladder angiogenesis and hyperplasia [79]. Host tissue repair responses have been described as a worm survival strategy for promoting the shedding of eggs and guaranteeing their dispersion into the environment [65,78].

*S. haematobium* ESP (*Sh*-ESP) have been demonstrated to have carcinogenic and diagnostic properties (Table S1) [76,80]. *Sh*-ESP induce rapid uncontrolled division, high resistance to death, and abnormal cell migration properties [81], and accelerate tumor development [82] and dysplasia in mice, suggesting that *Sh*-ESP induce pre-neoplastic lesions that can lead to cancer [83]. A major protein secreted by *S. haematobium* eggs is the ortholog of interleukin-4-inducing principle (IPSE) [80], which stimulates the secretion of interleukin 4 (IL-4) and interleukin 13 (IL-13) from basophils and mast cells by engaging IgE bound to the IgE receptor of these cells [84]. The *S. haematobium* orthologs H03-H-IPSE and H06-H-IPSE induce the proliferation of mouse urothelial cells and are internalized by urothelial and neuronal cells, promoting procarcinogenic programs [78].

### 3.4. Other Helminths Associated with Cancer in Humans and Animals

Other helminths have been associated with neoplasia in humans and animals without being formally classified as carcinogens by the IARC. Comprehensive studies are required on the prevalence of cancer-related infections and the secretome that may be involved.

#### 3.4.1. *Taenia solium*

*Taenia solium,* the pig tapeworm, causes neurocysticercosis in its intermediate hosts. Humans become infected when eggs of the parasite are ingested from contaminated food or water and develop cysticerci in different organs of the body [85]. Infection with *T. solium* has been associated with progression to glioblastoma multiforme, a neoplasm of the central nervous system [86]. However, a direct link between *T. solium* ESP and the development of cancer is still missing, even though the analysis of the genomic secretome of *T. solium* revealed that 10 proteins might be involved in cancer-associated pathways [87,88].

Studies in patients with neurocysticercosis have demonstrated that the production of nitric oxide and other ROS released from neutrophils and lymphocytes damage the host cell’s DNA, leading to genetic instability and chromosome aberrations, such as chromosome and chromatid breaks, as well as balanced and unbalanced translocations [86,89]. Chronic inflammation and unstable DNA in the peripheral lymphocytes derived from *T. solium* infection may lead to immunosuppression due to the reduced capacity of the lymphocytes to divide [90] and eventually contribute to the development of malignancies [86,90,91]. 

When the cysticercus is established in the central nervous system, molecules that regulate the host’s immune system are secreted. For instance, paramyosin and taeniaestatin affect complement activation with a subsequent downregulation of leukocyte function through the inhibition of C1 and depression of circulating complement levels [92,93]. In addition, the metacestode factor (MF) reduces the generation of antibodies against the parasite and inhibits lymphocyte proliferation and the production of interleukin 2 (IL-2), IL-4 and IFN-γ in stimulated splenocytes and of TNF-α in macrophages [94]. These mechanisms might contribute to the dysregulation of the host immune response, which may allow the transformation of astrocytes into malignant glial cells [88,89,94].

#### 3.4.2. *Echinococcus granulosus*

Cystic echinococcosis occurs in humans when *Echinococcus granulosus* eggs are ingested, leading to the formation of hydatid cysts in various tissues, mainly in the abdominal and chest cavities [95]. The role of the dog tapeworm *E. granulosus* in the development of cancer is still not well understood [60]. A positive statistical association with cancer was found in patients with colon, skin, breast and prostate cancer, years after the diagnosis of cystic echinococcosis [96]. Another study determined that *E. granulosus* protoscoleces modulate the host immune response and generate a suppressive microenvironment [97], with reductions in IFN-γ + and C-C chemokine receptor type 5 (CCR5+) Th1 cells and an increase in CD4+ CD25+ T cells compared to control animals [97]. 

Although several studies have found an association between *E. granulosus* and cancer, others have found a contrasting protective effect of the worms on malignancy development [98], with patients presenting a lower incidence of cancer compared to individuals without this worm infection [99,100]. It is hypothesized that this parasite elicits a protective effect against cancer due to antigenic similarities between components of hydatid cysts and cancer cells [100]. Moreover, in the acute and chronic phases of infection, oncospheres and hydatid cysts release Kunitz-type protease inhibitors (EgKI-1) and B antigen (Table S1), which inhibit neutrophil chemotaxis, and neutrophil-associated elastase, which affects the cell cycle [60,101,102]. Furthermore, the release of mucin-type O-glycan activates the innate and Th1 responses against cancer [60,100]. 

#### 3.4.3. *Strongyloides stercoralis*

The widespread nematode *Strongyloides stercoralis* causes intestinal infections known as strongyloidiasis in humans and other mammal hosts. Neoplasia development involves restorative hyperplasia due to the direct mucosal damage caused by the adult stages, eggs and, possibly, some secreted products of the parasite [103]. Chronic infection with this nematode in the gut and colon is related to gastrointestinal and colorectal cancer [103,104], and strongyloidiasis in the biliary intestinal tract may promote pancreatic and hepatic carcinogenesis [105]. The prevalence of *S. stercoralis* infections was higher in patients with cancer compared to control groups of infected individuals without cancer, with no significant association with a specific type of malignancy [106]. 

#### 3.4.4. *Heterakis gallinarum*

The cecal nematode of poultry *Heterakis gallinarum* can induce the formation of granulomatous nodules that can evolve to neoplasia classified as leiomyomas, cecal wall perforation and peritonitis, as a response to reinfections with different parasite strains from other domestic birds. [107,108]. As in the case of *T. solium*, further evidence is required to assess the potential role of ESP in the cellular transformation observed [107]. 

#### 3.4.5. *Trichuris muris*

Chronic infections with *Trichuris muris*, a natural gut-dwelling nematode of mice and the laboratory infection model for the human intestinal parasite *Trichocephalus trichiurus*, produce changes in the caeca of mice leading to the development of intestinal neoplasia [109]. Mouse cells infected with *T. muris* show increased inflammatory infiltrate in the lamina propria and epithelial hyperplasia, as well as high levels of IL-6, TNF-α and IFN-γ [110]. 

## 4. Possible Model of Cancer Induction in Esophageal Spirocercosis

Several hypotheses have been raised to explain the transformation of *S. lupi*-induced esophageal nodules to sarcomas. This process can be triggered by chronic inflammation, the constant repair of the tissue due to direct mechanical damage induced by the worms, or *Sl*-ESP [29,111]. Chronic inflammation during spirocercosis is characterized by neutrophil infiltration around the parasites, generating oxygen and nitric oxide reactive species involved in the oxidation of the host cell´s DNA. This can lead to chromosomal aberrations and subsequent malignant transformation, as previously suggested [29]. An increased mutation rate precedes alterations in oncogenes and tumor suppressor genes, which in combination with prosurvival signals, the inhibition of apoptosis and the stimulation of proliferation pathways induced by *Sl*-ESP such as galectin, 14-3-3 protein, HSP70 and HSP90, may promote neoplastic nodule formation. 

**Figure 2:**
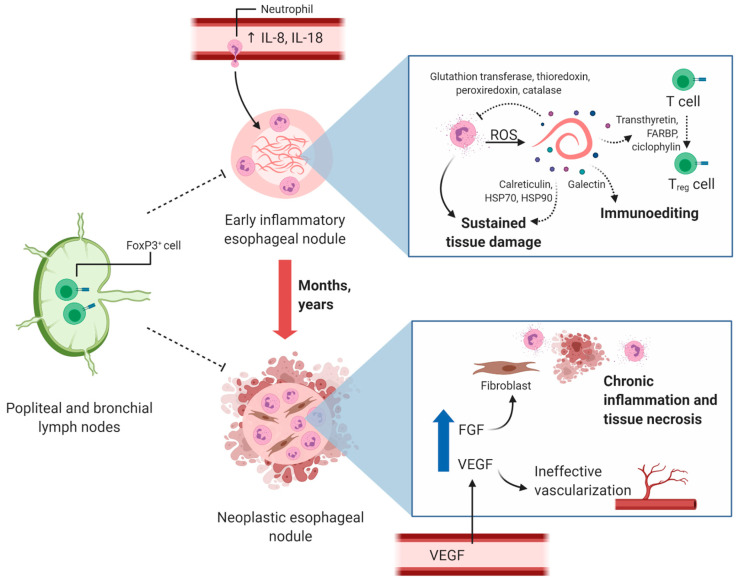
Proposed mechanism of cancer induction during *S. lupi* infection. High levels of blood IL-8 and IL-18 lead to neutrophil infiltration into esophageal nodules and sustained inflammation targeted at *S. lupi* worms. Adult *S. lupi* stages release products through excretion–secretion that have been shown to be involved in reactive-oxygen species (ROS) scavenging and Treg cell differentiation in other helminth parasites. The presence of *S. lupi* in nodules for months to years may lead to chronic tissue inflammation and neoplasia characterized by neutrophil infiltration and tissue necrosis. Increased vascular endothelial growth factor (VEGF) may have different roles in these lesions since low microvessel density is detected in neoplastic nodules. High fibroblast growth factor (FGF) may induce the differentiation of tissue-resident fibrocytes to fibroblasts and eventually to fibrosarcoma. Levels of FoxP3+ cells homing from popliteal and bronchial lymph nodes to esophageal nodules are decreased compared to what is commonly found in other malignancies and should be further studied. The potential role of some *S. lupi*-associated excretion–secretion products is depicted with dashed arrows. This figure was created using Biorender.com.

A strong inflammatory response might occur during the pre-neoplastic phase, with the recruitment and activation of myeloid cells, followed by immunoediting processes. Accordingly, the increased serum and plasma levels of IL-8 and IL-18 in dogs with early-inflammatory nodules could explain neutrophil chemotaxis to tissues and the induction of an inflammatory milieu. This, in turn, might trigger the production of more IL-8, leading to a positive feedback loop and sustained inflammation and tissue damage, affected by the reduced concentrations of the proinflammatory cytokines IL-18 and granulocyte-macrophage colony stimulating factor (GM-CSF), the latter being lower in the neoplastic group compared to the non-neoplastic, although not significantly different (Figure 2) [22]. The increased plasma concentrations of IL-18 in dogs with non-neoplastic nodules might reflect the characteristic inflammatory responses of pre-neoplastic lesions associated with neutrophil recruitment, free-radical production and cellular transformation. Moreover, IL-18 may induce antitumor immunity in combination with other immune mediators such as interleukin 12 (IL-12) and GM-CSF [112]. Additionally, IL-8 has been linked to tumor transdifferentiation through epithelial-to-mesenchymal transition, in which cells acquire mesenchymal characteristics, increase metastasis and favor an immunosuppressive microenvironment [113]. This immunosuppressive microenvironment might play a key role in the development of neoplastic lesions and might be induced by IL-8 and other tolerogenic mediators in response to chronic inflammation. Additionally, immunomodulatory molecules present in the *Sl*-ESP such as galectin might contribute to the immunoediting process, since members of this family of proteins have been classified as soluble immune checkpoints important in cancer immune regulation [114]. Even though these immunomodulatory events are expected in the development of the tumor microenvironment, further investigation is necessary to confirm the mechanisms involved in *S. lupi*-associated neoplastic nodule formation. 

*S. lupi*-derived ESP such as annexin 6, peroxiredoxin and thioredoxin might affect host-cell homeostasis and increase the risk of malignant transformation [111,115], based on observations from other helminths [68] and the role of some of these proteins in human carcinomas [116]. Annexin 6 has exhibited opposing effects in various types of cancer in humans, depending on the affected tissue and degree of malignancy, and has been associated with the deregulation of the rat sarcoma (Ras), Ras/mitogen-activated protein kinase (MAPK) and focal adhesion kinase (FAK)/phosphatidylinositol 3-kinase (PI3K) signaling pathways, which affect the cell cycle, adhesion, motility and invasiveness [116]. Nevertheless, the precise role of annexin 6 in *S. lupi*-induced oncogenesis should be studied further, as this molecule has not been investigated in other parasitic worms. The ROS-scavenging proteins thioredoxin and peroxiredoxin have demonstrated specific roles in the induction of cell transformation in host biliary cells by *O. viverrini* [67,68]. A similar mechanism might occur in early-inflammatory and pre-neoplastic nodules during esophageal spirocercosis, in which *S. lupi*-derived thioredoxin or peroxiredoxin may inhibit cell apoptosis induced by neutrophil oxidative stress.

Other proteins found in *Sl*-ESP and also in other nematode species not associated with cancer might play a role in cancer development. Calreticulin, HSP70 and HSP90 are translocated to the cell surfaces of stressed or dying tumor human cells, where they interact with antigen-presenting cells and induce cell maturation and the activation of a “danger” response characterized by the production of proinflammatory cytokines [38,39]. Calreticulin acts as an “eat me” signal for phagocytes, increasing the processing and presentation of tumor-associated and tumor-specific antigens, whereas cell surface HSP70 and HSP90 expression plays a major role in the cross-presentation of tumor-derived antigenic peptides, leading to specific T-cell responses [38,39]. Therefore, these proteins are considered adjuvants for the antitumor stimulation of immune responses. Regarding *Sl*-ESP proteins, it is important to consider that extracellular HSPs and calreticulin have context-dependent inflammatory or anti-inflammatory functions [117]. Therefore, in pre-neoplastic nodules, these proteins can activate the production of immune mediators, promoting chronic inflammation and tissue damage, whereas during the malignant phase, extracellular HSPs and calreticulin might dampen effective antitumor immune responses by affecting tumor antigen cross-presentation. The immunomodulatory protein galectin is associated with enhanced oncogenic signals, the regulation of tumor cell growth or apoptosis, the modulation of cell migration and the suppression of immune responses in human cancer [118], and may play a similar role in *S. lupi* tumors. *Sl*-ESP with intracellular oncogenic potential, 14-3-3 protein, elongation-factor-1 alpha, HSP70 and HSP90 are associated with increased survival, proliferation and apoptosis inhibition in several types of human cancer [39,40,41]. The potential role of these proteins in the development of malignant nodules during spirocercosis depends on the level of cellular internalization of the proteins or delivery via microvesicles and requires further investigation. 

Some helminths and their associated ESP affect immune surveillance and might contribute to the clonal expansion of transformed cells [112]. The cross-presentation of tumor antigenic peptides is necessary to establish an effective antitumor immune response. The cell surface protein chaperones calreticulin, HSP70 and HSP90 function as adjuvants in this process [39]. *S. lupi*-associated soluble chaperones might affect this process by saturating receptors important for the cellular interaction in cross-presentation and activating antigen-presenting cells in the absence of tumor-specific peptides. A Treg/Th17 imbalance that plays a role in the decreased immunosurveillance and favors cancer was described in *C. sinensis* infection [119]. However, FoxP3+ cells, characteristic of Treg responses, are not abundant in early-inflammatory, pre-neoplastic or neoplastic nodules in spirocercosis. Furthermore, the ESP transthyretin, retinoid fatty acid binding proteins and cyclophilin have been shown to be involved in the induction of Treg cells in *S. mansoni* infections, and these molecules were detected in all *S. lupi* stages, even though Treg cells are not abundant in the nodules [32,120]. Additionally, a higher number of FoxP3+ cells has been detected in the popliteal and bronchial lymph nodes of dogs with esophageal spirocercosis (Figure 2), suggesting a possible alteration of local immune responses that hinders these cells from homing to the nodules [29]. As previously mentioned, this can be explained by the low vascularization present in neoplastic nodules, another subpopulation of Treg cells without FoxP3+ [29], and the involvement of suppressor myeloid cells in the generation of the immunosuppressive microenvironment. Therefore, it is important to conduct further research to characterize the immune infiltrates of *S. lupi* nodules in all the stages, in detail.

## 5. Future Directions in *Spirocerca lupi* Proteomic Studies

*S. lupi* is well recognized as a cause of malignant esophageal neoplasms in dogs and the only nematode described to induce malignant processes in dogs [21]. For this reason, it has previously been suggested to use this parasite as a model to study the role of nematodes and their proteins as carcinogenic agents [115]. However, maintaining the life cycle of this parasite in definitive canid hosts is cumbersome. The use of common laboratory animals such as mice, rats and rabbits would not allow the replication of the life cycle because these animals act as paratenic hosts of the parasite [7]. L3 encysts in the gastric walls of these paratenic hosts and does not develop any further [9]. Moreover, maintaining *S. lupi* adults from naturally infected dogs is equally challenging due to the limited ability to keep them viable for long periods of time. One study kept adult *S. lupi* alive for only four days after harvest from the host [34], while in another study, female and male *S. lupi* adults were maintained for 36 and 16 days, respectively [35]. These technical obstacles should not relegate research on this intriguing parasite, and instead, the role of *Sl*-ESP in host–pathogen interactions should be studied, focusing on host cell transformation and immunoregulation. This will improve our understanding of the pathology induced by this parasite that leads to neoplastic transformation and may also aid in the prevention of malignancies in *S. lupi*-infected dogs around the world. 

## Supplementary Materials

The following are available online at https://www.mdpi.com/2076-0817/10/2/124/s1, Table S1: Proteins of parasitic helminths with a potential role in cancer development.
