# Supplementary material for: Spirocerca lupi Proteomics and Its Role in Cancer Development: An Overview of Spirocercosis-Induced Sarcomas and Revision of Helminth-Induced Carcinomas
Source: Pathogens. 2021 Jan 26;10(2):124. doi: 10.3390/pathogens10020124 (PMC7911836; doi:10.3390/pathogens10020124)
Supplement: Supplementary file 1 [file pathogens-10-00124-s001.pdf]

Table S1. Proteins of parasitic helminths with a potential role in cancer development.

Protein name (Abbreviation)	Parasite	Stage	Experiment	Effect	Reference
C. sinensis ferritin heavy chain (*Cs*FHC)	*C. sinensis*	Adult, metacercariae and eggs	*In vitro*	Proinflammatory mediator through the NF-κB signaling pathway.	[49]
C. sinensis granulin (*Cs*GRN)	*C. sinensis*	Adult	*In vivo*	Angiogenesis, insensitivity to apoptosis, tumor invasion and anchorage dependence	[59]
Group III secretory phospholipase A2 (*Cs*GIIIsPLA2)	*C. sinensis*	Adult	*In vitro*	Increase the production of type III collagen, hepatic fibrosis	[46]
C. sinensis lysophospholipase (*Cs*lysoPLA)	*C. sinensis*	Adult, metacercariae* and eggs	*In vitro, in vivo*	Development of hepatic fibrosis	[47]
C. sinensis fructose-1,6-bisphosphatase (*Cs*FBPase)	*C. sinensis*	Adult, metacercariae*	*In vitro*	Upregulation of key fibrosis-related factors	[48]
C. sinensis TFG-beta receptor interacting protein 1 (*Cs*TRIP1)	*C. sinensis*	Adult	*In vitro*	Modulator in the TGF-β signaling pathway	[55]
C. sinensis legumain (*Cs*Leg)	*C. sinensis*	Adult	*In vitro*	Cysteine protease activity	[55]
C. sinensis growth factor binding protein 2 (*Cs*Grb2)	*C. sinensis*	Adult	*In vitro*	Adaptor protein in the Ras-mediated signal transduction pathways	[55]
Annexin B30	*C. sinensis*	Adult, metacercariae*	*In vivo*	Triggers IL-10 production in splenocytes, might affect immune response during infection	[58, 121]
O. viverrini granulin-like growth factor (*Ov*-GRN-1)	*O. viverrini*	Adult	*In vitro*	Cell proliferation, angiogenesis, suppression of anoikis and tumor progression	[64, 122, 123].
Thioredoxin	*O. viverrini*	Adult	*In vitro*	Downregulates apoptotic genes, upregulates antiapoptosis-associated genes	[68]
Peroxiredoxin	*O. viverrini*	Adult, metacercariae and eggs	*In vitro*	Inhibits apoptosis, protection of parasite against ROS	[64, 124]
S. haematobium total antigen (Sh)	*S. haematobium*	Adult	*In vitro, In vivo*	Cell proliferation, resistance to apoptosis, migration, activating mutations of KRAS gene	[83, 125]
Interleukin-4-inducing principle (IPSE)	*S. haematobium*	Eggs	*In vivo*	Induction of proliferation	[78]
Kunitz-type protease inhibitor (EgKI-1)	*E. granulosus*	Oncosphere	*In vitro*	Inhibition of growth and migration of cancer cells by inducing apoptosis	[102]

Antigen B (AgB)	*E. granulosus*	Hydatid cyst	*In vitro*	Inhibition of neutrophil elastase and neutrophil chemotaxis, decreased apoptosis	[60, 101]
Mucin-type O-glycan	*E. granulosus*	Hydatid cyst	*In vitro*	Innate and Th-1 immune responses activation through immunological cross-reaction.	[60, 100]

*Secretion of the protein is higher in this stage.

121. Da Costa, J.M.C.; Vale, N.; Gouveia, M.J.; Botelho, M.C.; Sripa, B.; Santos, L.L.; Santos, J.H.; Rinaldi, G.; Brindley, P.J.; Da Costa, J.M.C.; et al. Schistosome and liver fluke derived catechol-estrogens and helminth associated cancers. *Front. Genet.* **2014**, *5*, doi:10.3389/fgene.2014.00444.
122. Smout, M.; Laha, T.; Mulvenna, J.; Sripa, B.; Suttiprapa, S.; Jones, A.; Brindley, P.J.; Loukas, A. A Granulin-Like Growth Factor Secreted by the Carcinogenic Liver Fluke, Opisthorchis viverrini, Promotes Proliferation of Host Cells. *PLoS Pathog.* **2009**, *5*, e1000611, doi:10.1371/journal.ppat.1000611.
123. Arunsan, P.; Ittiprasert, W.; Smout, M.; Cochran, C.J.; Mann, V.H.; Chaiyadet, S.; E Karinshak, S.; Sripa, B.; Young, N.D.; Sotillo, J.; et al. Programmed knockout mutation of liver fluke granulin attenuates virulence of infection-induced hepatobiliary morbidity. *eLife* **2019**, *8*, doi:10.7554/elife.41463.
124. Suttiprapa, S.; Loukas, A.; Laha, T.; Wongkham, S.; Kaewkes, S.; Gaze, S.; Brindley, P.J.; Sripa, B. Characterization of the antioxidant enzyme, thioredoxin peroxidase, from the carcinogenic human liver fluke, Opisthorchis viverrini. *Mol. Biochem. Parasitol.* **2008**, *160*, 116–122, doi:10.1016/j.molbiopara.2008.04.010.
125. Botelho, M.C.; Veiga, I.; Oliveira, P.A.; Lopes, C.; Teixeira, M.; da Costa, J.M.; Machado, J.C. Carcinogenic ability of *Schistosoma haematobium* possibly through oncogenic mutation of KRAS gene. *Adv. Cancer Res. Treat.* **2013**, *2013*, 876585.
